# Toxin-like peptides in plasma, urine and faecal samples from COVID-19 patients

**DOI:** 10.12688/f1000research.54306.2

**Published:** 2021-10-14

**Authors:** Carlo Brogna, Simone Cristoni, Mauro Petrillo, Maddalena Querci, Ornella Piazza, Guy Van den Eede

**Affiliations:** 1 Craniomed group srl, Montemiletto, 83038, Italy; 2 ISB Ion Source & Biotechnologies srl, Italy, Bresso, Milano, 20091, Italy; 3 European Commission, Joint Research Centre (JRC), Ispra, 21027, Italy; 4 Department of Medicine and Surgery, University of Salerno, Baronissi, 84081, Italy; 5 European Commission, Joint Research Centre (JRC), Geel, 2440, Belgium

**Keywords:** SARS-CoV-2, COVID-19, toxin-like peptides

## Abstract

**Background:** SARS-CoV-2 that causes COVID-19 disease and led to the pandemic currently affecting the world has been broadly investigated. Different studies have been performed to understand the infection mechanism, and the involved human genes, transcripts and proteins. In parallel, numerous clinical extra-pulmonary manifestations co-occurring with COVID-19 disease have been reported and evidence of their severity and persistence is increasing. Whether these manifestations are linked to other disorders co-occurring with SARS-CoV-2 infection, is under discussion. In this work, we report the identification of toxin-like peptides in COVID-19 patients by application of the Liquid Chromatography Surface-Activated Chemical Ionization – Cloud Ion Mobility Mass Spectrometry.

**Methods:** Plasma, urine and faecal samples from COVID-19 patients and control individuals were analysed to study peptidomic toxins’ profiles. Protein precipitation preparation procedure was used for plasma, to remove high molecular weight proteins and efficiently solubilize the peptide fraction; in the case of faeces and urine, direct peptide solubilization was employed.

**Results:** Toxin-like peptides, almost identical to toxic components of venoms from animals, like conotoxins, phospholipases, phosphodiesterases, zinc metal proteinases, and bradykinins, were identified in samples from COVID-19 patients, but not in control samples.

**Conclusions:** The presence of toxin-like peptides could potentially be connected to SARS-CoV-2 infection. Their presence suggests a possible association between COVID-19 disease and the release in the body of (oligo-)peptides almost identical to toxic components of venoms from animals. Their involvement in a large set of heterogeneous extra-pulmonary COVID-19 clinical manifestations, like neurological ones, cannot be excluded. Although the presence of each individual symptom is not selective of the disease, their combination might be related to COVID-19 by the coexistence of the panel of the here detected toxin-like peptides. The presence of these peptides opens new scenarios on the aetiology of the COVID-19 clinical symptoms observed up to now, including neurological manifestations.

## Introduction

Numerous clinical extra-pulmonary manifestations co-occurring with COVID-19 disease have been reported (e.g. neurological, haemorrhagic, and thrombotic) and evidence of their severity and persistence is increasing. Gupta *et al*. reviewed the extrapulmonary organ-specific pathophysiology of patients with COVID-19, ' *to aid clinicians and scientists in recognizing and monitoring the spectrum of manifestations, and in developing research priorities and therapeutic strategies for all organ systems involved*' ^1^. Liotta *et al*. characterized the incidence of neurological manifestations in a cohort of hospitalised patients with confirmed COVID-19: the most frequent were myalgia, headache, encephalopathy, dizziness, dysgeusia, and anosmia; encephalopathy was found to be ' *associated with increased morbidity and mortality, independent of respiratory disease severity*' ^2^. Whether these manifestations are linked to disorders co-occurring with SARS-CoV-2 infection is under discussion, including their concomitant occurrence, which could be strongly related COVID-19 disease. Frontera *et al*., by conducting a prospective, multi-centre, observational study of hospitalised adults with laboratory-confirmed SARS-CoV-2 infection, concluded that *'neurologic disorders were detected in 13.5% of COVID-19 patients during the study timeframe. Many of these neurologic disorders occur commonly among patients with critical illness. Encephalitis, meningitis or myelitis referable to SARS-CoV-2 infection did not occur, though post-infectious Guillain-Barre syndrome was identified. Overall, neurologic disorders in the context of SARS-CoV-2 infection confer a higher risk of in-hospital mortality and reduced likelihood of discharge home*' ^3^.

Studies on the use of mass spectrometry in COVID-19 context focus on the search for augmented human inflammatory molecules to be used as biomarkers to assess the severity status of COVID-19 (see for example the work ^4^ of Messner and colleagues). Different studies report the use of proteomic approaches to characterise SARS-CoV-2 proteins ^5– 7^. Other studies highlight challenges in their use due to the need of enriching the protein fraction to be analysed for maximizing the technology sensitivity ^8^.

 Liquid Chromatography Surface-Activated Chemical Ionization – Cloud Ion Mobility Mass Spectrometry (LC-SACI-CIMS) is reported as a high sensitivity mass spectrometry technique able to maximize the peptide signal intensity ^9– 12^. We used LC-SACI-CIMS to reveal the presence of metabolites that could explain the clinical descriptions of neurological, coagulation and inflammatory symptoms, and here we present the results of our analyses. We found toxin-like peptides in plasma, urine, and faecal samples from COVID-19 patients, but not in control samples. As our findings do not correspond with current thinking of the aetiology related to the observed clinical manifestations in COVID-19 patients, we feel their immediate sharing with the scientific community is critical.

## Methods

### Rationale

Liquid Chromatography-Surface Activated Chemical Ionization – Cloud Ion Mobility Mass Spectrometry (LC-SACI-CIMS) exhibits a high selectivity in peptide detection thanks to its ability to selectively isolate peptide ions through an in-source ion mobility (IM) effect. In fact, it allows a selective regulation of the potential difference between the low voltage of the SACI surface (47 V) and the entrance lens (-50 / -600 V), and a selective focalization on solvent ion cloud containing species at low or high *m/z* ratio. By switching the entrance voltage lens between -50 and -600 V during the analysis, it is possible to separate the low *m/z* from the high *m/z* potential signal, to avoid ion trap saturation, and to maximize the number of detected compounds. The mass spectra chemical noise is also strongly reduced due to the lower amounts of solvent cluster ions that are produced in low voltage ionization conditions. Thus, the peptide detection efficiency is strongly increased by the IM selectivity and lower chemical noise with respect to the classical high voltage ionization approaches. Thanks to the specificity of the SACI-CIMS technology in focalizing the solvent ion clouds containing the high *m/z* (oligo-)peptide species, it was possible to increase the detection efficiency.

In the use of LC-SACI-CIMS, the following strategies have been adopted:

- To reduce the presence of contamination as much as possible and to avoid the formation of acetonitrile polymers occurring in acid conditions (as reported by Eizo *et al*. ^13^), formic acid was not added to the CH _3_CN chromatographic phase.
- To separate low from high *m/z* solvent ion clusters by reducing the ion trap saturation, the space/charge effect, and by increasing the detected compounds recovery, LC-SACI–CIMS entrance lens voltage was switched between -50 and -600 V every 10 ms during the analysis.
- To enhance the SACI ionization efficiency, NH _4_HCO _3_ was added to the samples. As reported in the literature ^14, 15^, the peptide ionization efficiency (and consequently the sensitivity) is enhanced in SACI conditions when ionic salts are present in the sample, due to peptide ion specific coordination.
- To decrease the total run time, a shot gun chromatographic gradient was used to desalt the sample.
- To avoid sample molecular profile alteration, and to evaluate the potential biological activities of the circulating species, no enzymatic digestion was applied to samples.
- To normalize the *m/z* signal intensity, 5 µL of standard ESI tune mix (Agilent, USA) were added to each sample extract.

### Chemicals

NH _4_HCO _3_, methanol, acetonitrile and formic acid were purchased from Sigma-Aldrich (Milan, Italy). Bi-distilled water was purchased from VWR (Milan, Italy).

### Cohort

Samples used in the present study: plasma samples collected from 20 COVID-19 patients from different cities of Italy and from 10 control individuals (i.e. negative to SARS-CoV-2 tests and not affected by cancer or autoimmune diseases); urine samples collected from two additional COVID-19 patients and from two control individuals; stool samples from three COVID-19 patients and from three control individuals. The human biological samples used in the experimentation were collected and used with the expressed free and informed written consent, of the person from whom the material was taken, according to current legislation. The study received approval from “Comitato Etico Campania Sud” (n.36/2021, request submitted on 06-05-2020). Apart from positivity to SARS-CoV-2, no additional information (i.e. age, sex, blood serotype, severity of the disease, time of the collection, fatality, etc.) was provided.

### Sample preparation

***Plasma.*** Each plasma sample was treated as follows: 5 µL of CH _3_CN were added to 50 µL of plasma and vortexed for one minute. The procedure was repeated 10 times. Then the sample was centrifuged at 1,500 g for 10 minutes and two 100 µL aliquots of supernatant were dried and resuspended in 70 µL of NH _4_HCO _3_ 50 mmol. The solution was analysed by LC-SACI-CIMS (see *Rationale*).

***Urine.*** Each urine sample was treated as follows: an equivalent volume of bi-distilled water was added, followed by centrifugation at 1,500 g for 10 minutes. 100 µL were dried and resuspended in 70 µL of NH _4_HCO _3_ 50 mmol. The sample was analysed by LC-SACI-CIMS (see *Rationale*).

***Stool.*** Each stool sample was treated as described by Cristoni *et al*. ^11^ and analysed by LC-SACI-CIMS (see *Rationale*).

### Liquid chromatography

The Ultimate 3000 LC (by ThermoFisher) was used to achieve separation of analytes for each sample prior to mass spectrometry (MS) analysis. A reversed phase Kinetex C-18 LC column (50 × 2.1 mm; particle size, 5 µm; pore size, 100 Å, by Phenomenex, USA) was used. The eluent flow was 0.25 mL/min and the injection volume was 15 µL. The mobile phases were:

**A**. 0.2% (v/v) formic acid (HCOOH)
**B**. acetonitrile (CH _3_CN)

The elution gradient was: 2% (v/v) of B between 0 and 2 min; 2 to 30% between 2 and 7 min; 30 to 80% between 7 and 9 min; 80% between 9 and 12 min; 80-2% between 12 and 12.1 min. The column was rebalanced with 2% of B between 12.1 and 17 min.

### Mass spectrometry

All samples were analysed for the presence of proteins with potential toxic effect by using the LC-SACI-CIMS as already described in the literature ^9– 12^. Samples were analysed with an ORBITRAP mass spectrometer (Breme, Germany) coupled to a surface-activated chemical ionization (SACI) source and operated in positive ion mode.

The surface voltage was 47 V and the entrance lens was switched between -50 and -600 V each 10 ms. Auxiliary gas: 2 L / min; Nebulizer gas: 80 psi; Temperature: 40 °C. Full scan spectra were acquired in the 40–3,500 *m/z* range for non-targeted metabolomics/proteomics analyses to detect analytes. The same *m/z* range was used for both discovery and selective biomarker identification, and to standardize (primarily in terms of scan rate) the instrument. The software used for data elaboration is SANIST, a modified version of the Global Proteome Machine (GPM, https://www.thegpm.org/GPM/), implanted as described in 9– 12. SANIST output files are available as supplementary material ^16^ (see section *Data availability*).

SANIST software here used is freely available, upon email request to CranioMed group ( dir.brogna@craniomed.it).

Mass spectrometry on samples was performed with collision-induced dissociation using data dependent scan and helium as the collision gas. The ion trap was applied to isolate and fragment the precursor ions (windows of isolation, ± 0.3 *m/z*; collision energy, 30% of its maximum value, which was 5V peak to peak), and the ORBITRAP mass analyser was used to obtain fragments with an extremely accurate *m/z* ratio (resolution 15,000; *m/z* error <10 ppm).

### Data elaboration

Detected high *m/z* peptides were used to identify toxins thanks due to the selectivity given by their long chain. 

The complete UniprotKB set of manually reviewed venom proteins and toxins (UniprotKB, Animal toxin annotation project. https://www.uniprot.org/program/Toxins, Accessed October 4, 2020), mixed with a subset of non-venom proteins and toxins from UniprotKB database ^17^ was used as reference protein dataset in order to give statistical significance to the results.

TBLASTN ^18^ was run at the National Center for Biotechnology Information (NCBI) website ^19^ with default options and parameters, with the exception of the following ones: max target sequences = 1,000; expect threshold = 100; word size = 3; gap cost existence = 9; gap cost extension = 1; filter of low complexity regions = No. Searches have been performed versus: Nucleotide collection (nr/nt); Reference RNA sequences (refseq_rna); RefSeq Genome Database (refseq_genomes); Whole-genome shotgun contigs (wgs) from metagenomic experiments; Sequence Read Archive (SRA) sequences from metagenomic experiments; Transcriptome Shotgun Assembly (TSA); Patent sequences (pat); Human RefSeqGene sequences (RefSeq_Gene); Betacoronavirus Genbank sequence dataset.

The information reported in Table 1 has been retrieved from the UniprotKB database and from the NCBI Taxonomy database ^20^, after confirmation by BLAST sequence comparison analysis ^18^.

**Table 1.** Overview of candidate proteins on which toxin-like peptides have been mapped. Thirty-six candidate protein sequences on which the identified toxin-like peptides have been mapped are here reported, together with information retrieved from UniprotKB and NCBI Taxonomy databases. The table is split in three sections according to the phylum of the reported species: *Chordata* (green), *Echinodermata* (pink), *Mollusca* (azure).

UNIPROTKB CANDIDATE'S INFORMATION							TAXONOMY CANDIDATE'S INFORMATION						
*AC*	*ID*	*Status*	*Protein name*	*ENZYME EC*	*Other name(s)*	*Length (aa)*	*ID*	*Species*	*Phylum - Family*	*Organism's common name(s)*
Q8AY46	VKTHB_BUNCA	reviewed	Kunitz-type serine protease inhibitor homolog beta- bungarotoxin B1 chain	NA	-	85	92438	*Bungarus Candidus*	***Chordata*** - *Elapidae*	. Malayan krait
A6MEY4	PA2B_BUNFA	reviewed	Basic phospholipase A2 BFPA	EC 3.1.1.4	. Antimicrobial phospholipase A2 . Phosphatidylcholine 2-acylhydrolase (svPLA2)	145	8613	*Bungarus fasciatus*	***Chordata*** - *Elapidae*	. Banded krait . Pseudoboa fasciata
F5CPF1	PA235_MICAT	reviewed	Phospholipase A2 MALT0035C	EC 3.1.1.4	. Phospholipase A2 MALT0035C (svPLA2)	142	129457	*Micrurus altirostris*	***Chordata*** - *Elapidae*	. Uruguayan coral snake . Elaps altirostris
A8QL59	VM3_NAJAT	reviewed	Zinc metalloproteinase-disintegrin-like NaMP	EC 3.4.24.-	. Snake venom metalloproteinase (SVMP)	621	8656	*Naja atra*	***Chordata*** - *Elapidae*	. Chinese cobra
Q9I900	PA2AD_NAJSP	reviewed	Acidic phospholipase A2 D	EC 3.1.1.4	. svPLA2 . APLA . Phosphatidylcholine 2-acylhydrolase	146	33626	*Naja sputatrix*	***Chordata*** - *Elapidae*	. Malayan spitting cobra . Naja naja sputatrix
Q58L90	FA5V_OXYMI	reviewed	Venom prothrombin activator omicarin-C non-catalytic subunit	NA	. vPA . Venom coagulation factor Va-like protein *Cleaved into 2 chains*	1460	111177	*Oxyuranus microlepidotus*	***Chordata*** - *Elapidae*	. Inland taipan . Diemenia microlepidota
Q58L91	FA5V_OXYSU	reviewed	Venom prothrombin activator oscutarin-C non-catalytic subunit	NA	. vPA . Venom coagulation factor Va-like protein *Cleaved into 2 chains*	1459	8668	*Oxyuranus scutellatus*	***Chordata*** - *Elapidae*	. Coastal taipan
Q9W7J9	3S34_PSETE	reviewed	Short neurotoxin 4	NA	. SNTX4 . Alpha-neurotoxin 4	79	8673	*Pseudonaja textilis*	***Chordata*** - *Elapidae*	. Eastern brown snake
P23028	PA2AD_PSETE	reviewed	Acidic phospholipase A2 homolog textilotoxin D chain	NA	. svPLA2 homolog	152	8673	*Pseudonaja textilis*	***Chordata*** - *Elapidae*	. Eastern brown snake
Q593B6	FA5_PSETE	reviewed	Coagulation factor V	NA	*Cleaved into 2 chains*	1459	8673	*Pseudonaja textilis*	***Chordata*** - *Elapidae*	. Eastern brown snake
Q7SZN0	FA5V_PSETE	reviewed	Venom prothrombin activator pseutarin-C non-catalytic subunit	NA	. PCNS . vPA . Venom coagulation factor Va-like protein *Cleaved into 2 chains*	1460	8673	*Pseudonaja textilis*	***Chordata*** - *Elapidae*	. Eastern brown snake
Q2XXQ3	CRVP1_PSEPL	reviewed	Cysteine-rich venom protein ENH1	NA	. CRVP . Cysteine-rich secretory protein ENH1 (CRISP-ENH1)	239	338839	*Pseudoferania polylepis*	***Chordata*** - *Homalopsidae*	. Macleay's water snake . Enhydris polylepis
Q9PW56	BNP2_BOTJA	reviewed	Bradykinin-potentiating and C-type natriuretic peptides	NA	. Brain BPP-CNP . Evasin-CNP *Cleaved into the 12 chains*	265	8724	*Bothrops jararaca*	***Chordata*** - *Viperidae*	. Jararaca
A8YPR6	SVMI_ECHOC	reviewed	Snake venom metalloprotease inhibitor	NA	. 02D01 . 02E11 . 10F07 . Svmpi-Eoc7 *Cleaved into 15 chains*	308	99586	*Echis oceIIatus*	***Chordata*** - *Viperidae*	. Ocellated saw-scaled viper
Q698K8	VM2L4_GLOBR	reviewed	Zinc metalloproteinase/disintegrin [Fragment]	EC 3.4.24-	*Cleaved into 3 chains*	319	259325	*Gloydius brevicaudus*	***Chordata*** - *Viperidae*	. Korean slamosa snake . Agkistrodon halys brevicaudus
Q8AWI5	VM3HA_GLOHA	reviewed	Zinc metalloproteinase-disintegrin-like halysase	EC 3.4.24-	. Zinc metalloproteinase-disintegrin-like halysase . Snake venom metalloproteinase (SVMP) . Vascular apoptosis-inducing protein (VAP)	610	8714	*Gloydius halys*	***Chordata*** - *Viperidae*	. Chinese water mocassin . Agkistrodon halys
P82662	3L26_OPHHA	reviewed	Alpha- neurotoxin	NA	. Alpha-elapitoxin-Oh2b (Alpha-EPTX-Oh2b) . Alpha-elapitoxin-Oh2b . LNTX3 . Long neurotoxin OH-6A/OH-6B . OH-3	91	8665	*Ophiophagus hannah*	***Chordata*** - *Viperidae*	. King cobra . Naja hannah
Q2PG83	PA2A_PROEL	reviewed	Acidic phospholipase A2 PePLA2	EC 3.1.1.4	. Phosphatidylcholine 2-acylhydrolase (svPLA2)	138	88086	*Protobothrops elegans*	***Chordata*** - *Viperidae*	. Elegant pitviper . Trimeresurus elegans
P06860	PA2BX_PROFL	reviewed	Basic phospholipase A2 PL-X	EC 3.1.1.4	. Phosphatidylcholine 2-acylhydrolase (svPLA2)	122	88087	*Protobothrops flavoviridis*	***Chordata*** - *Viperidae*	. Habu . Trimeresurus flavoviridis
P0C7P5	BNP_PROFL	reviewed	Bradykinin-potentiating and C-type natriuretic peptides	NA	. BPP-CNP *Cleaved into 6 chains*	193	88087	*Protobothrops flavoviridis*	***Chordata*** - *Viperidae*	. Habu . Trimeresurus flavoviridis
Q3C2C2	PA21_ACAPL	reviewed	Phospholipase A2 AP-PLA2T	EC 3.1.1.4	. Phosphatidylcholine 2-acylhydrolase (svPLA2)	159	133434	*Acanthaster planci*	***Echinodermata*** - *Acanthasteridae*	. Crown-of-thorns starfish
D6C4M3	CU96_CONCL	reviewed	Conotoxin Cl9.6	NA	. Conotoxin CI9.6	81	1736779	*Californiconus californicus*	***Mollusca*** - *Conidae*	. California cone - Conus californicus
D2Y488	VKT1A_CONCL	reviewed	Kunitz-type serine protease inhibitor conotoxin Cal9.1a	NA	-	78	1736779	*Californiconus californicus*	***Mollusca*** - *Conidae*	. California cone . Conus californicus
D6C4J8	CUE9_CONCL	reviewed	Conotoxin Cl14.9	NA	-	78	1736779	*Californiconus californicus*	***Mollusca*** - *Conidae*	. California cone . Conus californicus
P0DPT2	CA1B_CONCT	reviewed	Alpha- conotoxin ClB [Fragment]	NA	. C1.2	41	101291	*Conus catus*	***Mollusca*** - *Conidae*	. Cat cone
V5V893	CQG3_CONFL	reviewed	Conotoxin Fla16d	NA	. Conotoxin Flal6d *Cleaved into 2 chains*	76	101302	*Conus flavidus*	***Mollusca*** - *Conidae*	. Yellow Pacific cone
P58924	CS8A_CONGE	reviewed	Sigma- conotoxin GVIIIA	NA	. Sigma-conotoxin GVIIIA	88	6491	*Conus geographus*	***Mollusca*** - *Conidae*	. Geography cone . Nubecula geographus
P0DM19	NF2_CONMR	reviewed	Conotoxin Mr15.2	NA	. Conotoxin Mr15.2 (Mr094)	92	42752	*Conus marmoreus*	***Mollusca*** - *Conidae*	. Marble cone
P0C1N5	M3G_CONMR	reviewed	Conotoxin mr3g	NA	. Conotoxin mr3g (Mr3.6)	68	42752	*Conus marmoreus*	***Mollusca*** - *Conidae*	. Marble cone
D2DGD8	I361_CONPL	reviewed	Conotoxin Pu6.1	NA	-	83	93154	*Conus pulicarius*	***Mollusca*** - *Conidae*	. Flea-bite cone
P0C8U9	CA15_CONPL	reviewed	Alpha- conotoxin-like Pu1.5	NA	-	81	93154	*Conus pulicarius*	***Mollusca*** - *Conidae*	. Flea-bite cone
A1X8B8	CAl_CONQU	reviewed	Putative alpha- conotoxin Qc alphaL-1	NA	. QcaL-1	68	101313	*Conus quercinus*	***Mollusca*** - *Conidae*	. Oak cone
P58786	COW_CONRA	reviewed	Contryphan-R	NA	. Bromocontryphan *Cleaved into 2chains*	63	61198	*Conus radiatus*	***Mollusca*** - *Conidae*	. Rayed cone
P58811	CA1A_CONTU	reviewed	Rho- conotoxin TIA	NA	. Rho-TIA	58	6495	*Conus tulipa*	***Mollusca*** - *Conidae*	. Fish-hunting cone snail . Tulip cone
Q5K0C5	016A_CONVR	reviewed	Conotoxin 10	NA	-	79	89427	*Conus virgo*	***Mollusca*** - *Conidae*	. Virgin cone
B3FIA5	CVFA_CONVR	reviewed	Conotoxin Vi15a	NA	. Conotoxin Vi15.l	74	8765	*Conus virgo*	***Mollusca*** - *Conidae*	. Virgin cone

SANIST was set to perform the database search considering all potential protein points and post-translational modifications, and to consider proton rearrangements. No enzyme cutting rules were specified, but all the protein subsequence combinations were considered. Database search calculation was performed by means of General Processing Graphic Processing Units (GPGPU).

The MS data are available on the ZENODO platform ^16^ (see section *Data availability*).

## Results and discussion

**Figure 1.**
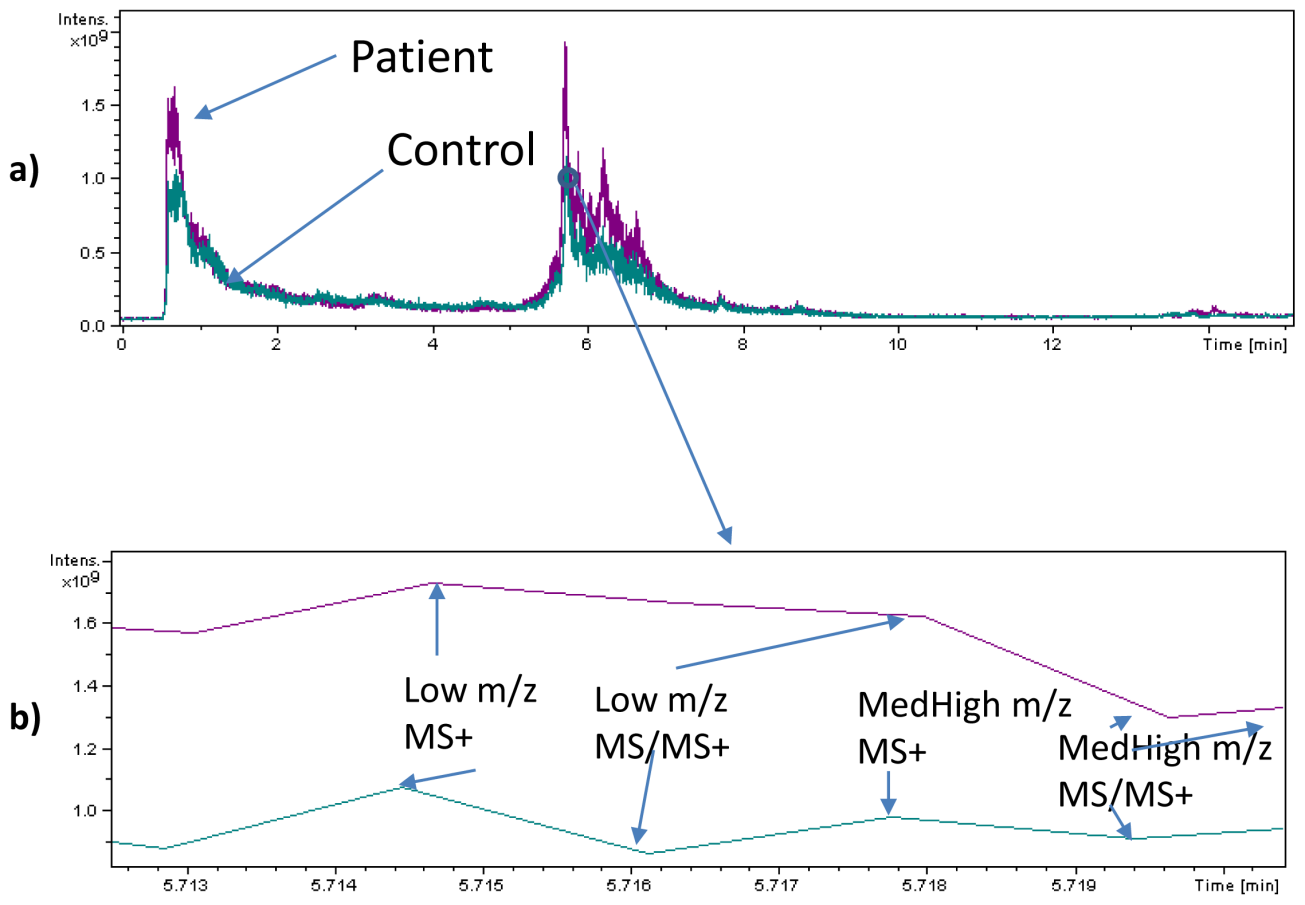
( **a**) Base peak LC Full Scan (MS), tandem mass (MS/MS) chromatogram of an extracted plasma sample of a patient and a control subject and ( **b**) a blow-up of a specific chromatogram region (5.713–5.719 min). The blow-up shows the four regions of data acquisition: 1) Full scan mass spectrum originated by the cloud containing low *m/z* ratio molecular species; 2) Tandem mass spectra (MS/MS) mass spectrum originated by the cloud containing low *m/z* ratio molecular species; 3) Full scan mass spectrum originated by the cloud containing medium-high (MedHigh) *m/z* ratio molecular species; 4) Tandem mass spectra (MS/MS) mass spectrum originated by the cloud containing medium-high (MedHigh) *m/z* ratio molecular species.

**Figure 2.**
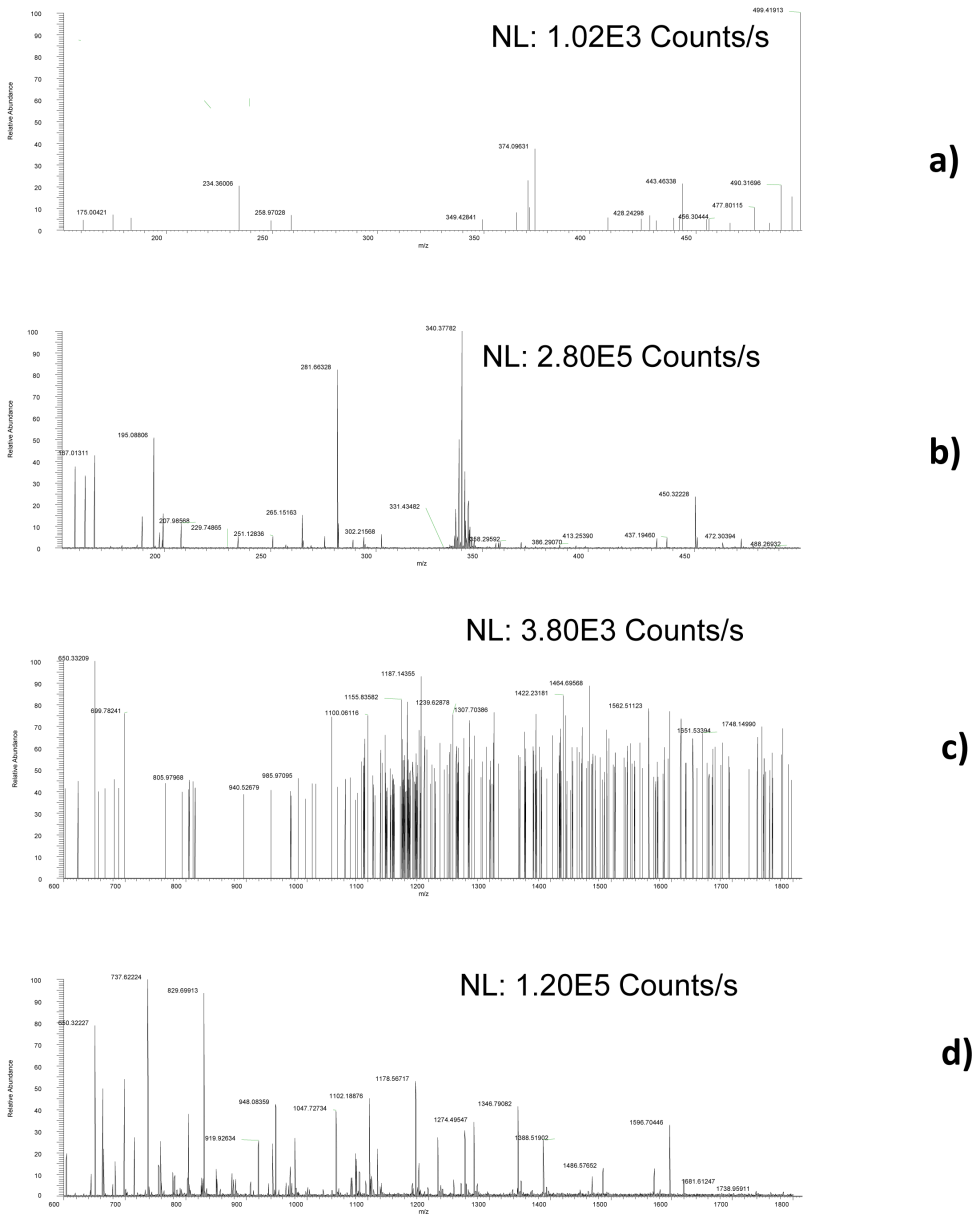
Examples of full scan mass spectra, obtained by analysing a COVID-19 positive urine sample and acquired focalizing solvent ion cloud species containing **a**) low, **b**) high *m/z* species extracted in the 5.713–5.719 min chromatographic region and ESI full scan mass spectrum obtained analysing the same sample and extracting the signal at the same retention time extracting **c**) low and **d**) high m/z ratio.

 The presence of (oligo-)peptides characterised as toxic components of animal venoms was observed in plasma and urine samples from SARS-CoV-2 infected patients and never in plasma, urine and faecal samples from control individuals. Examples of SACI-CIMS chromatograms are reported in Figure 1 and Figure 2 (panels a and b), showing the spectra acquired by means of the LC-SACI-CIMS technology. Figure 2c and d show the spectra obtained using ESI extracted at the same retention time. SACI-CIMS give rise to higher signal intensities probably due to the low ion trap saturation.

**Figure 3.**
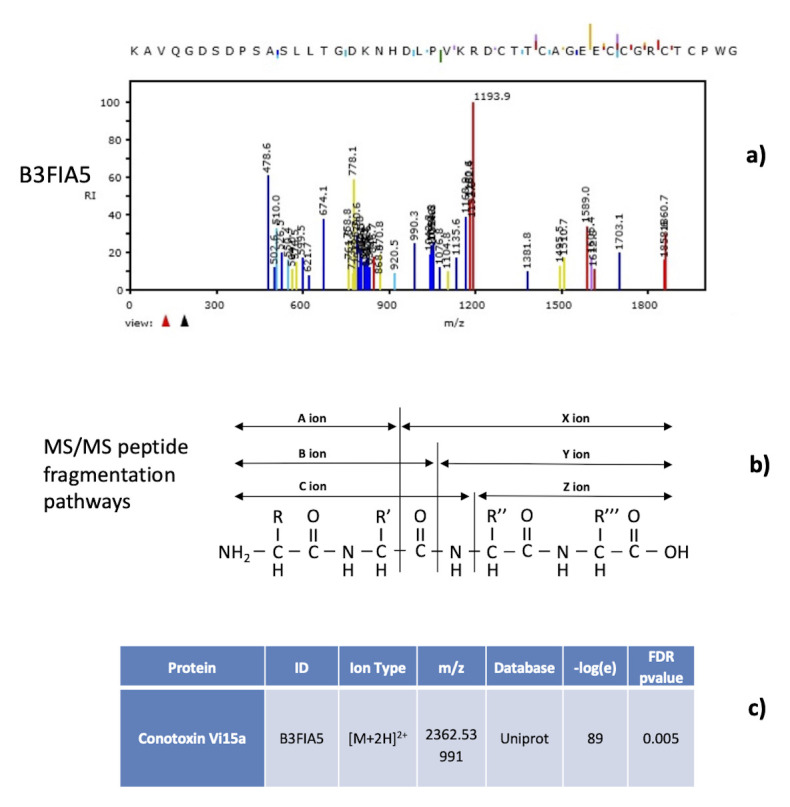
Examples of mass spectra peptide characterization together with the peptide ion fragmentation pathways. Example of how MS/MS signal were assigned to the different N-terminal y,z (blue and purple colour in panel *a*) and c-terminal b,c (red and yellow colour) fragmentation series (detailed in panel *b*). Only mass spectra exhibiting a statistical -log(e) score higher that 10 and a false discovery rate lower than 0.05 were considered for the identification (reported in panel *c*). False discovery rate and statistical score were estimated by means of reverse sequence approach.

Several (oligo-)peptides (between 70 and 115, depending on the analysed sample) matched to different animal venom proteins and toxins like conotoxins, phospholipases A2, metalloproteinases (86% of assignments have a *-log(e)* higher than 25). An overview of 36 proteins covered by the toxin-like peptides found is reported in Table 1; details of -log(e) and false discovery rates are reported in Table 2. Examples of mass spectra peptide characterization together with the peptide ion fragmentation pathways are shown in Figure 3a. All the MS/MS signal were assigned to the different N-terminal y,z (blue and purple colour) and c-terminal b,c (red and yellow colour) fragmentation series (see Figure 3b for fragmentation series details). In the defined SACI-CIMS conditions, doubly charged *m/z* ion of medium-high molecular weight peptide species are produced, allowing high identification accuracy, in line with what is already described in the literature that high identification statistical rates are achieved analysing peptide doubly charged species with medium high molecular weight. Different fragmentation anomalies with proton rearrangements have also been detected and considered in phase of data elaboration. Only mass spectra exhibiting a statistical - *log(e)* score higher that 10 and a false discovery rate lower than 0.05 were considered for the identification (see Figure 3c). False discovery rate and statistical score were estimated by means of reverse sequence approach.

**Table 2.** List of proteins and the related -log(e) and false discovery ratio (FDR) expressed as p value.

Protein	ID	Database	-log(e)	FDR p value
Conotoxin Pu6.1	D2DGD8	Uniprot	75	0.001
Conotoxin Vi15a	B3FIA5	Uniprot	89	0.005
Putative alpha-conotoxin Qc alphaL-1	A1X8B8	Uniprot	76	0.005
Conotoxin 10	Q5K0C5	Uniprot	76	0.001
Rho-conotoxin TIA	P58811	Uniprot	54	0.001
Kunitz-type serine protease inhibitor conotoxin Cal9.1a	D2Y488	Uniprot	67	0.001
Alpha-conotoxin Pu1.5	P0C8U9	Uniprot	57	0.002
Conotoxin Fla16d	V5V893	Uniprot	67	0.003
Phospholipase A2 MALT0035C	F5CPF1	Uniprot	87	0.003
Phospholipase A2 AP-PLA2-I	Q3C2C2	Uniprot	81	0.004
Acidic phospholipase A2 PePLA2	Q2PG83	Uniprot	66	0.001
Basic phospholipase A2 BFPA	A6MEY4	Uniprot	69	0.001
Basic phospholipase A2 PL-X	P06860	Uniprot	70	0.001
Complement factor B Ba fragment	Q91900	Uniprot	74	0.001
Acidic phospholipase A2 homolog textilotoxin D chain	P23028-1	Uniprot	73	0.002
Acidic phospholipase A2 homolog textilotoxin D chain	P23028-2	Uniprot	65	0.002
Venom prothrombin activator pseutarin-C non-catalytic subunit	Q7SZN0	Uniprot	60	0.002
Coagulation factor V	Q593B6	Uniprot	61	
Venom prothrombin activator oscutarin-C non-catalytic subunit	Q58L91	Uniprot	87	0.001
Short neurotoxin 4	Q9W7J9	Uniprot	69	0.001
Conotoxin Cl9.6	D6C4M3	Uniprot	58	0.002
Zinc metalloproteinase-disintegrin-like halysase	Q8AWI5	Uniprot	57	0.003
Alpha-elapitoxin-Oh2b	P82662	Uniprot	96	0.003
Sigma-conotoxin GVIIIA	P58924	Uniprot	43	0.002
Conotoxin Mr15.2	P0DM19	Uniprot	47	0.001
Conotoxin mr3g	P0C1N5	Uniprot	74	0.001
Contryphan-R	P58786	Uniprot	58	0.002
Snake venom metalloprotease inhibitor 02D01	A8YPR6	Uniprot	43	0.002
Bradykinin-potentiating and C-type natriuretic peptides	P0C7P5	Uniprot	51	0.003
Bradykinin-potentiating and C-type natriuretic peptides	Q9PW56	Uniprot	51	0.003
Zinc metalloproteinase/ disintegrin	Q698K8	Uniprot	49	0.004

**Figure 4.**
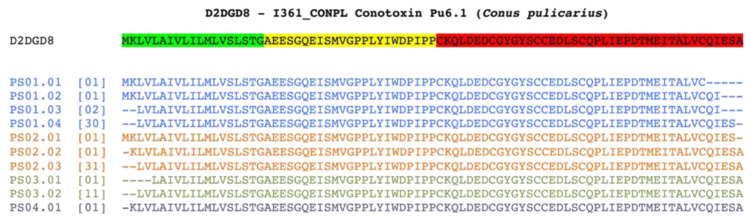
Alignment of toxin-like peptides to Conotoxin Pu6.1 precursor. Conotoxin Pu6.1 precursor from *Conus pulicarius* (UniprotKB:D2DGD8) is aligned with the toxin-like peptides identified in four out of five plasma samples. Being the protein secreted and cleaved, leader-region pro-peptide and mature cysteine rich domains are highlighted in green, yellow and red, respectively. The shown peptides correspond to the longest observed peptides, as we did not make any specific selection for secreted proteins, precursors are expected to be present in our samples. Each identified toxin-like peptide is named according to the sample of origin and its uniqueness. For each of them, the number reported in square brackets indicates the number of identical toxin-like peptides identified in the same sample.

Some of the toxin-like peptides found mapped on the same reference protein (UniprotKB: D2DGD8), are reported in Figure 4: these peptides were found in the five plasma samples and in the three faecal samples.

The types of toxic-like peptides found resemble known conotoxins, phospholipases A2, metalloproteinases, prothrombin activators, coagulation factors, usually present in animal venoms, which are known to have high specificity and affinity towards human ion channels, receptors, and transporters of the nervous system, like the nicotinic acetylcholine receptor. Cheng *et al.* ^21^ reported the discovery of a superantigen-like motif in the S1 Spike protein, as well as two other neurotoxin-like motifs that have peptide similarities to neurotoxins from *Ophiophagus* (cobra) and Bungarus genera. They conclude that neurotoxin-like motifs are present in SARS-CoV-2 protein products, acting as neurotoxin-like peptides. We checked in the full set of peptides we got (here we report only 36 examples), and we identified, in plasma and faecal samples, toxin-like peptides mapping on kappa 1a-bungarotoxin, Kappa 1b-bungarotoxin from Malayan krait, kappa-2-bungarotoxin and alpha-bungarotoxin from many-banded krait (Uniprot Accession Numbers Q8AY56, Q8AY55, P15816, and P60615, respectively), which were reported by Cheng and colleagues. Furthermore, we looked at the amino acid changes currently reported in GISAID data ^22^, analysed by CoV-GLUE-Viz (update 15/09/2021) ^23^, and occurring in the Y _674_QTQTNSPRRAR _685_ motif identified by these authors as homologous to neurotoxin motifs of animal venom proteins. We observed the existence of amino acid variations which makes this motif even more similar to the neurotoxin motifs of animal venom proteins (like variations Q677S and T676A observed in sequences assigned to PANGO Lineage B.1.596). Experiments to assess neurotoxicity of these peptides and of spike protein on 3D neuronal/glial model (“neurospheres”) obtained from human induced Pluripotent Stem Derived Neural Stem Cells (iPS-NSCs) are currently ongoing.

What follows is our attempt to elaborate a potential relation between their presence and extra-pulmonary COVID-19 symptomatology.

### Conotoxins

Conotoxins are neurotoxic peptides isolated from the venom of marine (genus *Conus*) cone snails. In their mature form, they consist of 10 to 30 amino acid residues, with often one or more disulphide bonds, which are used to classify them in structural classes (μ-conotoxins, ω-conotoxins, and α-conotoxins are the major classes). The mechanism of action of conotoxins is not yet fully understood ^24^. Studies have found that they are able to modulate the activity of several receptors, including ion channels, nicotinic acetylcholine receptors (nAChRs) and acetylcholine-degrading enzymes (acetylcholinesterases), thus resulting in the alteration of acetylcholine levels and of cholinergic transmission ^25– 27^. Regarding cholinesterases, a potential association between cholinesterase levels and severity of pneumonia in COVID-19 patients has been proposed ^28^.

The presence of conotoxin peptides might explain the occurrence of many symptoms (like hyposmia, hypogeusia and the signs typical of Guillain-Barre syndrome) observed in some COVID-19 patients. Their presence can alter normal functioning of ion channels, nicotinic acetylcholine receptors and of acetylcholine levels.

### Phospholipases A2

Phospholipases A2 (PLA _2_, E.C. 3.1.1.4) hydrolyse phospholipids and lead to release of lysophosphatidic acid and arachidonic acid ^29^. Arachidonic acid is a major precursor of many pro-inflammatory mediators like leukotriene, thromboxane and prostaglandin; as a consequence, abnormal presence of active PLA _2_ can induce severe inflammation ^30^. In animal venoms, PLA _2 _act as neurotoxic proteins: they hydrolyse membrane phospholipids of the motor nerve terminal, and the plasma membrane of skeletal muscle, thus triggering a severe inflammatory degenerative response, which in turn leads to degeneration of the nerve terminal and skeletal muscle ^29^. The drug dexamethasone can inhibit prostaglandins synthesis and leukotriene formation ^31^. As dexamethasone is still the only therapeutic shown to be effective against the novel coronavirus in patients ^32^ with severe symptoms, it can be that the positive effect of this drug on COVID-19 patients is also due to the reduction of the here identified PLA _2_-like peptides.

### Metalloproteinases

The last example of identified toxin-like peptides is those recognised as metalloproteinases present in animal venoms, zinc-dependent enzymes of varying molecular weight having multidomain organization. These toxic enzymes cause haemorrhage, local myonecrosis, skin damage, and inflammatory reaction ^33^. It has been reported that symptomatic COVID-19 patients have significantly lower zinc levels in comparison to controls and that zinc deficient patients develop more complications ^34^. The presence of this specific group of toxin-like peptides, which capture zinc, can be one of the reasons for such significantly low zinc levels in symptomatic COVID-19 patients.

Similarity searches by TBLASTN ^14^ with relaxed parameters at the National Center for Biotechnology Information (NCBI) website (see Methods) revealed (in addition to mRNA sequences from the animal species reported in Table 1) almost identical short stretches (up to 10 amino acids) of these peptides in potential coding regions of many bacterial and viral sequences, but no long potential coding frame entirely covering any of them was found. Consequently, at the time of writing we have not yet identified the "genetic source" of these peptides, which could be:

- The SARS-CoV-2 RNA genome with its protein reading set, as proposed by Brogna ^35^, who reported the identification in SARS-CoV-2 RNA of many regions encoding for oligopeptides (four–five amino acids long) identical to neurotoxin peptides typical of animal venoms.
- The SARS-CoV-2 genome directly read by bacteria, assuming that the SARS-CoV-2 genome, or parts thereof, is capable of replicating with a possible ‘bacteriophage-like’ mode of action, as previously described ^36^.
- Genomes of bacteria, which, as a reaction to the presence of the virus, secrete these peptides. This could happen by using still not well known and debated mechanisms, like alternative reading due to rRNA sequence heterogeneity (as described in 37, 38), or the involvement of small bacterial ncRNA (sRNAs), known to be key players of gene regulation under conditions like stress response, quorum sensing, or virulence (in this context, in 1984 Coleman *et al*. reported the *micF* non-coding RNA as a functional bacterial sRNA ^39^).
- A combination of the above e.g. the ‘toxin’ genetic code is present in the bacteria and expression may be triggered by SARS-CoV-2, acting like temperate bacteriophages, which are known to interact with bacteria so that they express (or not) certain genes, as described by Carey *et al*. ^40^.

A detailed 3D structural similarity analysis between the toxin-like peptides found and reference proteins has not yet been conducted. Accordingly, at the time of writing, we can only speculate that these toxin-like peptides are involved in the clinical extra-pulmonary manifestations in symptomatic COVID-19 patients. According to our knowledge, these toxin-like peptides have never been searched in animals considered reservoirs of SARS-CoVs.

## Conclusions

The presence of (oligo-)peptides almost identical to toxic components of venoms from animals has been observed. Data and results reported here suggest an association between COVID-19 disease and the release in the body of these, and raise a series of questions:

- Are these findings in line with what was proposed by Tizabi *et al*. ^41^, i.e. a potential therapeutic role for nicotine, nicotinic agonists, or positive allosteric modulators of nicotinic cholinergic receptors in COVID-19?
- If induced by SARS-CoV-2, can the production of toxin-like peptides be involved in the neurological disorders and injuries observed in hospitalized COVID-19 patients?
- If induced by SARS-CoV-2, can the production of toxin-like peptides influence complex diseases apparently triggered or enhanced by COVID-19, like e.g. Guillain-Barré Syndrome ^42^ or Parkinson's disease ^43^?
- Are toxin-like peptides associated with SARS-CoV-2 infection or to other viral infections or, more in general, is their presence related to sickness condition?
- Are our findings supporting the suggestion made by the iVAMP Consortium ^44^ on the relationships between animal venom glands and microorganisms' microenvironments?

We consider that the immediate sharing of these results can contribute to the untangling of the multifaceted set of clinical manifestations in symptomatic COVID-19 patients, and to the further understanding of the mechanisms involved.

## Data availability

### Underlying data

Uniprot: Kunitz-type serine protease inhibitor homolog beta-bungarotoxin B1 chain [ *Bungarus candidus* (Malayan krait)]. Accession number Q8AY46, https://identifiers.org/uniprot:Q8AY46

Uniprot: Basic phospholipase A2 BFPA, svPLA2, EC 3.1.1.4 (Antimicrobial phospholipase A2) (Phosphatidylcholine 2-acylhydrolase) [ *Bungarus fasciatus* (Banded krait) (Pseudoboa fasciata)]. Accession number A6MEY4, https://identifiers.org/Uniprot:A6MEY4

Uniprot: Phospholipase A2 MALT0035C, svPLA2, EC 3.1.1.4 [ *Micrurus altirostris* (Uruguayan coral snake) (Elaps altirostris)]. Accession number F5CPF1, https://identifiers.org/Uniprot:F5CPF1

Uniprot: Zinc metalloproteinase-disintegrin-like NaMP, EC 3.4.24.- (Snake venom metalloproteinase, SVMP) [ *Naja atra* (Chinese cobra)]. Accession number A8QL59, https://identifiers.org/Uniprot:A8QL59

Uniprot: Acidic phospholipase A2 D, svPLA2, EC 3.1.1.4 (APLA) (Phosphatidylcholine 2-acylhydrolase) [ *Naja sputatrix* (Malayan spitting cobra) (Naja naja sputatrix)]. Accession number Q9I900, https://identifiers.org/Uniprot:A9I900

Uniprot: Venom prothrombin activator omicarin-C non-catalytic subunit, vPA (Venom coagulation factor Va-like protein) [Cleaved into: Omicarin-C non-catalytic subunit heavy chain; Omicarin-C non-catalytic subunit light chain] [ *Oxyuranus microlepidotus* (Inland taipan) (Diemenia microlepidota)]. Accession number A58L90, https://identifiers.org/Uniprot:Q58L90

Uniprot: Venom prothrombin activator oscutarin-C non-catalytic subunit, vPA (Venom coagulation factor Va-like protein) [Cleaved into: Oscutarin-C non-catalytic subunit heavy chain; Oscutarin-C non-catalytic subunit light chain] [ *Oxyuranus scutellatus* (Coastal taipan)]. Accession number Q58L91, https://identifiers.org/Uniprot:Q58L91

Uniprot: Short neurotoxin 4, SNTX4 (Alpha-neurotoxin 4) [ *Pseudonaja textilis* (Eastern brown snake)]. Accession number Q9W7J9, https://identifiers.org/Uniprot:Q9W7J9

Uniprot: Acidic phospholipase A2 homolog textilotoxin D chain, svPLA2 homolog [ *Pseudonaja textilis* (Eastern brown snake)]. Accession number P23028, https://identifiers.org/Uniprot:P23028

Uniprot: Coagulation factor V [Cleaved into: Coagulation factor V heavy chain; Coagulation factor V light chain] [ *Pseudonaja textilis* (Eastern brown snake)]. Accession number Q593B6, https://identifiers.org/Uniprot:Q593B6

Uniprot: Venom prothrombin activator pseutarin-C non-catalytic subunit, PCNS, vPA (Venom coagulation factor Va-like protein) [Cleaved into: Pseutarin-C non-catalytic subunit heavy chain; Pseutarin-C non-catalytic subunit light chain] [ *Pseudonaja textilis* (Eastern brown snake)]. Accession number Q7SZN0, https://identifiers.org/Uniprot:Q7SZN0

Uniprot: Cysteine-rich venom protein ENH1, CRVP (Cysteine-rich secretory protein ENH1, CRISP-ENH1) [ *Pseudoferania polylepis* (Macleay's water snake) (Enhydris polylepis)]. Accession number Q2XXQ3, https://identifiers.org/Uniprot:Q2XXQ3

Uniprot: Bradykinin-potentiating and C-type natriuretic peptides (Brain BPP-CNP, bBPP-CNP) (Evasin-CNP) [Cleaved into 12 chains] [ *Bothrops jararaca* (Jararaca)]. Accession number Q9PW56, https://identifiers.org/Uniprot:Q9PW56

Uniprot: Snake venom metalloprotease inhibitor 02D01 (02E11) (10F07) (Svmpi-Eoc7) [Cleaved into 15 chains] [ *Echis ocellatus* (Ocellated saw-scaled viper)]. Accession number A8YPR6, https://identifiers.org/Uniprot:A8YPR6

Uniprot: Zinc metalloproteinase/disintegrin [Cleaved into: Snake venom metalloproteinase brevilysin L4, SVMP (Snake venom metalloproteinase hxl-1, EC 3.4.24.-) ; Disintegrin brevicaudin-1a; Disintegrin brevicaudin-1b (Disintegrin adinbitor) (Disintegrin halystatin)] [Gloydius brevicaudus (Korean slamosa snake) (Agkistrodon halys brevicaudus)]. Accession number Q698K8, https://identifiers.org/Uniprot:Q698K8

Uniprot: Zinc metalloproteinase-disintegrin-like halysase, EC 3.4.24.- (Snake venom metalloproteinase, SVMP) (Vascular apoptosis-inducing protein, VAP) [ *Gloydius halys* (Chinese water mocassin) (Agkistrodon halys)]. Accession number Q8AWI5, https://identifiers.org/Uniprot:Q8AWI5

Uniprot: Alpha-elapitoxin-Oh2b, Alpha-EPTX-Oh2b (Alpha-neurotoxin) (LNTX3) (Long neurotoxin OH-6A/OH-6B) (OH-3) [ *Ophiophagus hannah* (King cobra) (Naja hannah)]. Accession number P82662, https://identifiers.org/Uniprot:P82662

Uniprot: Acidic phospholipase A2 PePLA2, svPLA2, EC 3.1.1.4 (Phosphatidylcholine 2-acylhydrolase) [ *Protobothrops elegans* (Elegant pitviper) (Trimeresurus elegans)]. Accession number Q2PG83, https://identifiers.org/Uniprot:Q2PG83

Uniprot: Basic phospholipase A2 PL-X, svPLA2, EC 3.1.1.4 (Phosphatidylcholine 2-acylhydrolase) [ *Protobothrops elegans* (Elegant pitviper) (Trimeresurus elegans)]. Accession number P06860, https://identifiers.org/Uniprot:P06860

Uniprot: Bradykinin-potentiating and C-type natriuretic peptides (BPP-CNP) [Cleaved into six chains] [ *Protobothrops flavoviridis* (Habu) (Trimeresurus flavoviridis)]. Accession number P0C7P5, https://identifiers.org/Uniprot:P0C7P5

Uniprot: Phospholipase A2 AP-PLA2-I, PLA2, EC 3.1.1.4 (Phosphatidylcholine 2-acylhydrolase 2) [ *Acanthaster planci* (Crown-of-thorns starfish)]. Accession number Q2C2C2, https://identifiers.org/Uniprot:Q3C2C2

Uniprot: Conotoxin Cl9.6 [ *Californiconus californicus* (California cone) (Conus californicus)]. Accession number D6C4M3, https://identifiers.org/Uniprot:D6C4M3

Uniprot: Kunitz-type serine protease inhibitor conotoxin Cal9.1a [ *Californiconus californicus* (California cone) (Conus californicus)]. Accession number D2Y488, https://identifiers.org/Uniprot:D2Y488

Uniprot: Conotoxin Cl14.9 [ *Californiconus californicus* (California cone) (Conus californicus)]. Accession number D6C4J8, https://identifiers.org/Uniprot:D6C4J8

Uniprot: Alpha-conotoxin CIB (C1.2) [ *Conus catus* (Cat cone)]. Accession number P0DPT2, https://identifiers.org/Uniprot:P0DPT2

Uniprot: Conotoxin Fla16d (Conotoxin Fla16.1) [Cleaved into: Conotoxin fla16a; Conotoxin fla16b; Conotoxin fla16c] [ *Conus flavidus* (Yellow Pacific cone)], Accession number V5V893, https://identifiers.org/Uniprot:V5V893

Uniprot: Sigma-conotoxin GVIIIA [ *Conus geographus* (Geography cone) (Nubecula geographus)]. Accession number P58924, https://identifiers.org/Uniprot:P58924

Uniprot: Conotoxin Mr15.2 (Mr094) [ *Conus marmoreus* (Marble cone)]. Accession number P0DM19, https://identifiers.org/Uniprot:P0DM19

Uniprot: Conotoxin mr3g (Mr3.6) [ *Conus marmoreus* (Marble cone)]. Accession number P0C1N5, https://identifiers.org/Uniprot: P0C1N5

Uniprot: Conotoxin Pu6.1 [ *Conus pulicarius* (Flea-bitten cone)]. Accession number D2DGD8, https://identifiers.org/Uniprot:D2DGD8

Uniprot: Alpha-conotoxin-like Pu1.5 [ *Conus pulicarius* (Flea-bitten cone)]. Accession number P0C8U9, https://identifiers.org/Uniprot:P0C8U9

Uniprot: Putative alpha-conotoxin Qc alphaL-1, QcaL-1 [ *Conus quercinus* (Oak cone)]. Accession number A1X8B8, https://identifiers.org/Uniprot:A1X8B8

Uniprot: Contryphan-R (Bromocontryphan) [Cleaved into: [Des-Gly1]-contryphan-R] [ *Conus radiatus* (Rayed cone)]. Accession number P58786, https://identifiers.org/Uniprot:P58786

Uniprot: Rho-conotoxin TIA, Rho-TIA [ *Conus tulipa* (Fish-hunting cone snail) (Tulip cone)]. Accession number P58811, https://identifiers.org/Uniprot:P58811

Uniprot: Conotoxin 10 [ *Conus virgo* (Virgin cone)]. Accession number Q5K0C5, https://identifiers.org/Uniprot:Q5K0C5

Uniprot: Conotoxin Vi15a (Vi15.1) [ *Conus virgo* (Virgin cone)]. Accession number B3FIA5, https://identifiers.org/Uniprot:B3FIA5

Zenodo: Underlying data for ‘Toxin-like peptides in plasma, urine and faecal samples from COVID-19 patients’, https://doi.org/10.5281/zenodo.4903154 ^16^

This project contains the following underlying data:

- Data file 1: Toxins.fasta
- Data file 2: Toxins.mgf

Data are available under the terms of the Creative Commons Attribution 4.0 International license (CC-BY4.0)

## Consent

The human biological samples used in the experimentation were collected and used with the expressed free and informed written consent of the person from whom the material was taken, according to current legislation.

## Declarations

The scientific output expressed does not imply a policy position of the European Commission. Neither the European Commission nor any person acting on behalf of the Commission is responsible for the use that might be made of this publication.
