# Supplementary material for: Toxin-like peptides in plasma, urine and faecal samples from COVID-19 patients
Source: F1000Res. 2021 Oct 14;10:550. Originally published 2021 Jul 8. [Version 2] doi: 10.12688/f1000research.54306.2 (PMC8772524; doi:10.12688/f1000research.54306.2)
Supplement: Peer review history

# Peer review: Reviewer response for version 2

**DOI:** 10.5256/f1000research.78337.r96937

**Published:** 2021-10-29

**Authors:** Moshe Arditi

**Affiliations:** 1 Department of Pediatrics, Division of Pediatric Infectious Diseases and Immunology, Cedars-Sinai Medical Center, Los Angeles, CA, USA

I approve the paper as it is now – the paper is excellent now.

Is the work clearly and accurately presented and does it cite the current literature?

Partly

If applicable, is the statistical analysis and its interpretation appropriate?

Yes

Are all the source data underlying the results available to ensure full reproducibility?

Partly

Is the study design appropriate and is the work technically sound?

Yes

Are the conclusions drawn adequately supported by the results?

Yes

Are sufficient details of methods and analysis provided to allow replication by others?

Yes

Reviewer Expertise:

NA

I confirm that I have read this submission and believe that I have an appropriate level of expertise to confirm that it is of an acceptable scientific standard.

# Peer review: Reviewer response for version 2

**DOI:** 10.5256/f1000research.78337.r96936

**Published:** 2021-10-18

**Authors:** Paolo Grumati

**Affiliations:** 1 Telethon Institute of Genetics and Medicine (TIGEM), Pozzuoli, Italy

Author addressed the requested points.

Is the work clearly and accurately presented and does it cite the current literature?

Yes

If applicable, is the statistical analysis and its interpretation appropriate?

No

Are all the source data underlying the results available to ensure full reproducibility?

Partly

Is the study design appropriate and is the work technically sound?

Partly

Are the conclusions drawn adequately supported by the results?

No

Are sufficient details of methods and analysis provided to allow replication by others?

Partly

Reviewer Expertise:

biology

I confirm that I have read this submission and believe that I have an appropriate level of expertise to confirm that it is of an acceptable scientific standard.

# Peer review: Reviewer response for version 1

**DOI:** 10.5256/f1000research.57783.r93677

**Published:** 2021-10-05

**Authors:** Moshe Arditi

**Affiliations:** 1 Department of Pediatrics, Division of Pediatric Infectious Diseases and Immunology, Cedars-Sinai Medical Center, Los Angeles, CA, USA

The investigators report the identification of toxin-like peptides in COVID-19 patients samples (Plasma, urine and stool samples) by using Liquid Chromatography Surface-Activated Chemical Ionization-Cloud Ion Mobility Mass Spectrometry. The investigators used a study cohort (for plasma) of 15 COVID 19 patients from different cities of Italy and from 5 control uninfected individuals. They collected urine samples from 2 COVID19 patients and 2 controls, and stool samples from 3 COVID19 patients and 3 controls. They report that toxin-like peptides, almost identical to toxic components such as conotoxins, phospholipases, phosphodiesterases etc. were identified from COVID19 patients, but not in any control samples.   They report an overview of 36 proteins covered by the toxin-like peptides they have found in plasma of COVID1- patients. These toxin-like peptides they discovered are very much like various neurotoxins, such as alpha Conotoxins, alpha Cobratoxins or similar to Bungarotoxins, all known to be neurotoxins.

 These are very important observations, the authors are asking the question is the COVID-19 infection is somehow inducing these toxin-like peptides in the host and if so, if these neurotoxin- like peptides maybe playing a functional role of the neurologic findings that are frequently associated with COVID-19 infection. One very important and potentially critical paper that must be mentioned in the discussion and that authors have missed was recently published by Mary Hongying Cheng *et al*.(2020 ^1^) where the investigators discovered a Superantigen-like motif in the S1 Spike protein, as well as two other neurotoxins that have peptide similarities to alpha cobratoxin and alpha bungarotoxin, alpha cobratoxin etc. Given this PNAS paper, it is now clear that the SARS -CoV2 virus contains neurotoxin-like peptides already. It would be incredibly interesting to see if the neurotoxin like peptides described and discovered in this PNAS paper are present in the toxin-like peptides described in this specific study. It will make this paper and its discussion much more impactful. At a minimum the PNAS paper should be discussed and cited.

Is the work clearly and accurately presented and does it cite the current literature?

Partly

If applicable, is the statistical analysis and its interpretation appropriate?

Yes

Are all the source data underlying the results available to ensure full reproducibility?

Partly

Is the study design appropriate and is the work technically sound?

Yes

Are the conclusions drawn adequately supported by the results?

Yes

Are sufficient details of methods and analysis provided to allow replication by others?

Yes

Reviewer Expertise:

Immunology, Infectious Diseases, Innate Immune Responses

I confirm that I have read this submission and believe that I have an appropriate level of expertise to confirm that it is of an acceptable scientific standard, however I have significant reservations, as outlined above.

## References

[1] : Superantigenic character of an insert unique to SARS-CoV-2 spike supported by skewed TCR repertoire in patients with hyperinflammation. *Proceedings of the National Academy of Sciences* .2020;117(41) : 10.1073/pnas.2010722117 25254-25262 10.1073/pnas.2010722117 PMC756823932989130

# Response

**Published:** 2021-10-05

**Authors:** Mauro Petrillo

**Affiliations:** European Commission, Joint Research Centre (JRC), Ispra, Italy

Dear Dr Arditi,

 thanks a lot for the valuable comments and suggestions that you have provided in the report.

 We will address all of them in order to provide a fully revised version of the manuscript.

 Best regards,

 Mauro Petrillo, on behalf of the authors.

# Response

**Published:** 2021-10-09

**Authors:** Mauro Petrillo

**Affiliations:** European Commission, Joint Research Centre (JRC), Ispra, Italy

Dear Dr Arditi,

 Thanks a lot for your valuable comments and suggestions that you have provided in the report. As anticipated, we have addressed all your points, and provided a new version of the manuscript:

- *One very important and potentially critical paper that must be mentioned in the discussion and that authors have missed was recently published by Mary Hongying Cheng et al.(2020) where the investigators discovered a Superantigen-like motif in the S1 Spike protein, as well as two other neurotoxins that have peptide similarities to alpha cobratoxin and alpha bungarotoxin, alpha cobratoxin etc. Given this PNAS paper, it is now clear that the SARS -CoV2 virus contains neurotoxin-like peptides already. It would be incredibly interesting to see if the neurotoxin like peptides described and discovered in this PNAS paper are present in the toxin-like peptides described in this specific study. It will make this paper and its discussion much more impactful. At a minimum the PNAS paper should be discussed and cited.*
- **Response**: We thank the Dr. Arditi for the comment and we fully agree with his suggestion to cite the Mary Hongying Cheng et al. (2020) PNAS paper. With respect to this point, we would like to highlight that the peptides reported in the paper as examples are a subset of more than 100 peptides identified by MS. We checked in the full set of peptides, and we can confirm that we identified, in plasma and faecal samples, toxin-like peptides mapping on kappa 1a-bungarotoxin, Kappa 1b-bungarotoxin from Malayan krait, kappa-2-bungarotoxin and alpha-bungarotoxin from many-banded krait (Uniprot Accession Numbers Q8AY56, Q8AY55, P15816, and P60615, respectively). To address this point, we added the following paragraph in the section “Results and discussion”: *Cheng et al. [REF] reported the discovery of a superantigen-like motif in the S1 Spike protein, as well as two other neurotoxin-like motifs that have peptide similarities to neurotoxins from Ophiophagus (cobra) and Bungarus genera. They conclude that neurotoxin-like motifs are present in SARS-CoV-2 protein products, acting as neurotoxin-like peptides. We checked in the full set of peptides we got (here we report only 36 examples), and we identified, in plasma and faecal samples, toxin-like peptides mapping on kappa 1a-bungarotoxin, Kappa 1b-bungarotoxin from Malayan krait, kappa-2-bungarotoxin and alpha-bungarotoxin from many-banded krait (Uniprot Accession Numbers Q8AY56, Q8AY55, P15816, and P60615, respectively), which were reported by Cheng and colleagues. Furthermore, we looked at the amino acid changes currently reported in GISAID data [REF], analysed by CoV-GLUE-Viz (update 15/09/2021) [REF], and occurring in the Y674QTQTNSPRRAR685 motif identified by these authors as homologous to neurotoxin motifs of animal venom proteins. We observed the existence of amino acid variations which makes this motif even more similar to the neurotoxin motifs of animal venom proteins (like variations Q677S and T676A observed in sequences assigned to PANGO Lineage B.1.596). Experiments to assess neurotoxicity of these peptides and of spike protein on 3D neuronal/glial model (“neurospheres”) obtained from human induced Pluripotent Stem Derived Neural Stem Cells (iPS-NSCs) are currently ongoing. What follows is our attempt to elaborate a potential relation between their presence and extra-pulmonary COVID-19 symptomatology.*

We hope that the quality of the manuscript, thanks to your comments, has been improved and you consider it suitable for indexing.

 Best regards,

 Mauro Petrillo, on behalf of the authors.

# Peer review: Reviewer response for version 1

**DOI:** 10.5256/f1000research.57783.r90696

**Published:** 2021-08-09

**Authors:** Paolo Grumati

**Affiliations:** 1 Telethon Institute of Genetics and Medicine (TIGEM), Pozzuoli, Italy

**12th August 2021: This peer review report was originally published with a Not Approved status, but the reviewer has since notified the Editorial team that this was not intended and it should be an Approved with Reservations. The report approval status has been updated to reflect this.**

 In the present manuscript the authors proposed an interesting consequence of COVID 19 infection. The idea is that COVID infection induces the production of toxins that are responsible for the specific clinical manifestations. Despite the fact that the origin of the toxins is not clear. The authors identified via mass spectrometry peptides that match the sequences of toxin components of venoms from animals. These observations are surprising and provocative. However, there are several points that the authors should consider:

1. COVID-19 outbreak is a pandemic therefore the number of affected people is extremely high. The number of samples analysed should be much higher. Authors should consider to have at least three different groups. Non-infected control, infected without clinical symptoms, affected with severe symptoms. For each group at least 10 samples for each analysis (blood, urine) should be analysed in order to have a more reliable statistic analysis. Moreover, does the amount of toxins correlate with the severity of the phenotype? 
2. Authors should provide some biological data that the toxins they identified are responsible for the clinical phenotype. They should perform some in vitro experiments infecting cells (CALU are the most used) with COVID or treating them with toxins. The outcome should be similar.
3. It is difficult to access the original data. It would be interesting to see the peptide sequences identified from the mass spectrometry. In Fig.4 the authors reported an example but it is unlike that a secreted toxin contains the leader region pro-peptide.
4. The text needs some editing. Citation of other research papers should be conform to the standard. 

Is the work clearly and accurately presented and does it cite the current literature?

Yes

If applicable, is the statistical analysis and its interpretation appropriate?

No

Are all the source data underlying the results available to ensure full reproducibility?

Partly

Is the study design appropriate and is the work technically sound?

Partly

Are the conclusions drawn adequately supported by the results?

No

Are sufficient details of methods and analysis provided to allow replication by others?

Partly

Reviewer Expertise:

biology

I confirm that I have read this submission and believe that I have an appropriate level of expertise to confirm that it is of an acceptable scientific standard, however I have significant reservations, as outlined above.

# Response

**Published:** 2021-08-12

**Authors:** Mauro Petrillo

**Affiliations:** European Commission, Joint Research Centre (JRC), Ispra, Italy

Dear Dr Grumati,

 Thanks a lot for your valuable comments and suggestions that you have provided in the report.

 We will address all of them, and wait for those of other reviewers, in order to provide a fully revised version of the manuscript.

 Best regards,

 Mauro Petrillo, on behalf of the authors.

# Response

**Published:** 2021-10-09

**Authors:** Mauro Petrillo

**Affiliations:** European Commission, Joint Research Centre (JRC), Ispra, Italy

Dear Dr Grumati,

 Thanks a lot for your valuable comments and suggestions that you have provided in the report. As anticipated, we have addressed all your points, and provided a new version of the manuscript:

- *COVID-19 outbreak is a pandemic therefore the number of affected people is extremely high. The number of samples analysed should be much higher. Authors should consider to have at least three different groups. Non-infected control, infected without clinical symptoms, affected with severe symptoms. For each group at least 10 samples for each analysis (blood, urine) should be analysed in order to have a more reliable statistic analysis. Moreover, does the amount of toxins correlate with the severity of the phenotype?*
- **Response: **We thank the Dr. Grumati for the comment. We increased the number of analysed plasma samples to address this point: in addition to the 5 control cases and 15 hospitalised cases (already mentioned in the section “Methods: Cohort”), we have analysed 5 plasma samples of infected individuals with mild or no symptoms, together with 5 additional controls. Thus, for plasma, there are now 30 cases (20 positive to SARS-CoV-2 tests plus 10 controls). In this new added group, we observe the presence of toxin-like peptides, apparently in lower amounts (in terms of *-loge*) with respect to the samples from hospitalised individuals. However, as we have no additional information about the grade of severity of the hospitalised subjects, we prefer to not infer any correlation between the amount of identified peptides and the severity of the phenotype. To better clarify this point, we have added the following sentence in the section “Methods:Cohort”: *Apart from positivity to SARS-CoV-2, no additional information (i.e. age, sex, blood serotype, severity of the disease, time of the collection, fatality, etc.) was provided.*
- *Authors should provide some biological data that the toxins they identified are responsible for the clinical phenotype. They should perform some in vitro experiments infecting cells (CALU are the most used) with COVID or treating them with toxins. The outcome should be similar.*
- **Response:** We thank the Dr. Grumati for the comment. Experiments to assess neurotoxicity of these peptides on 3D neuronal/glial model (“neurospheres”) obtained from human induced Pluripotent Stem Derived Neural Stem Cells (iPS-NSCs) are ongoing and will be part of an additional publication. We added a sentence in the section “Results and discussion” to address this point: *Experiments to assess neurotoxicity of these peptides on 3D neuronal/glial model (“neurospheres”) obtained from human induced Pluripotent Stem Derived Neural Stem Cells (iPS-NSCs) are ongoing.*
- *It is difficult to access the original data. It would be interesting to see the peptide sequences identified from the mass spectrometry. In Fig.4 the authors reported an example but it is unlike that a secreted toxin contains the leader region pro-peptide.*
- **Response: **We thank the Dr. Grumati for these comments. Regarding the data, we followed the journal policy: all data produced and here presented are freely and publicly available on the Zenodo platform ( http://www.doi.org/10.5281/zenodo.4903154, as reported in the section “Data availability” and in Reference 16, including MS files in MGF format and sequence files in FastA format). Regarding the alignment in Fig.4, the shown peptides correspond to the longest peptides we observed (LC-SACI-CIMS is particularly able in detect long peptides), aligned with respect to the precursor of the protein. We did not make any specific selection for secreted proteins; thus, precursors are expected to be present in our samples. To address this point, we modified the legend of Fig.4 accordingly.
- *The text needs some editing. Citation of other research papers should be conform to the standard.*
- **Response: **Thanks a lot for spotting these inconsistencies. We revised citations according to the journal specifications. We hope that the quality of the manuscript, thanks to your comments, has been improved and you consider it suitable for publication.

Best regards,

 Mauro Petrillo, on behalf of the authors.
